# Supplementary material for: Innate immune sensing via the cGAS-STING pathway restricts extrachromosomal DNA–driven tumorigenesis
Source: bioRxiv. 2026 Jan 1:2025.12.31.697191. Preprint. [Version 1] doi: 10.64898/2025.12.31.697191 (PMC12776550; doi:10.64898/2025.12.31.697191)
Supplement: 1 [file NIHPP2025.12.31.697191V1-supplement-1.pdf]

bioRxiv preprint doi: https://doi.org/10.64898/2025.12.31.697191; this version posted January 1, 2026. The copyright holder for this preprint (which was not certified by peer review) is the author/funder, who has granted bioRxiv a license to display the preprint in perpetuity. It is made available under aCC-BY 4.0 International license.

Supplementary Figure Legends

Figure S1. The cGAS-STING pathway is frequently defective in ecDNA$^+$ cancer cells. (**A**) mRNA levels of key components of nucleic acid sensing pathways in overlapping TCGA and PCAWG tumor samples with ecDNA (*upper-left*), with other focal amplifications—BFB, non-cyclic complex or linear amplification but without ecDNA (*upper-right*), and without detected focal amplifications (*bottom-left*). (*bottom-right*) All TCGA PANCAN samples subtract ecDNA$^+$ samples. (**B**) *cGAS* and *STING* mRNA levels in selected human cell lines. (**C**) The schematics show the location of the CpG rich region of human *STING* gene. The heatmaps show the DNA methylation levels of individual CpG sites as measured by bisulfite sequencing.

Figure S2. Immune activation by restoring the cGAS-STING pathway in human ecDNA$^+$ cancer cells. (**A&B**) SNU16 cells (ecDNA$^+$) were stably transduced with tetracycline-inducible *STING* constructs: wild-type (WT), the cGAMP-binding deficient mutant R238A/Y240A. After 0.1 μg/mL doxycycline induction for 24 hrs, *CXCL10* mRNA levels were measured by RT-qPCR (**A**) and STING protein levels determined by immunoblotting (**B**). (**C**) cGAMP levels in human BJ-5ta, THP-1, and COLO320DM cells induced to express cGAS^{WT} with doxycycline. (**D**) cGAMP levels in human glioblastoma cell lines GBM39KT (ecDNA$^+$) and GBM39HSR (ecDNA$^-$) expressing cGAS^{WT} or the inactive mutant cGAS^{AA} (G213A/S214A).

Figure S3. cGAS is activated by cytoplasmic ecDNA. (**A**) The workflow for isolating DNA from total or cytosolic lysate of COLO320DM and COLO320HSR cells, and for quantification of selected DNA targets. (**B**) Relative quantities of selected DNA sequences representing the nuclear genome (*B2M*), ecDNA (*MYC1* and *MYC2*), or mitochondria genome (*Dloop* and *ND1*) in fractions obtained in (A). Data are normalized to the total levels of nuclear target *KCNJ1*. (**C**) CIP2A was knocked out by Cas9-sgRNA in COLO320DM and COLO320HSR cells. cGAMP levels were quantified after inducing cGAS expression with doxycycline.

Figure S4. Isolation of an ecDNA$^+$ cell line from the *KPfC* mice with pancreatic adenocarcinoma. (A) The KPfC *Kras$^{LSL-G12D/+}$Trp53$^{fl/fl}$Pdx1$^{Cre/+}$* GEMM on the C57BL/6 background was generated by intercrossing *Kras$^{LSL-G12D/+}$Trp53$^{fl/fl}$* mice with Pdx1-Cre

bioRxiv preprint doi: https://doi.org/10.64898/2025.12.31.697191; this version posted January 1, 2026. The copyright holder for this preprint (which was not certified by peer review) is the author/funder, who has granted bioRxiv a license to display the preprint in perpetuity. It is made available under aCC-BY 4.0 International license.

transgenic mice. The dual alteration of *Kras* and *Trp53* drives spontaneous initiation and progression of pancreatic ductal adenocarcinoma (PDAC). A primary pancreatic cancer cell line CT1BA5 was isolated from late-stage tumor and subsequently subcloned to generate two clonal cell lines: CT1BA5-EC (ecDNA^{+}) and CT1BA5-HSR (focal amplification of *Kras* as HSR) (co-submitted manuscript by Qiao et al[35]). FISH in metaphase spread show focal amplification of *Kras*. (**B**) The upper schematic shows the location of the CpG island in the Exon 1 of murine *cGAS* gene. The bottom heatmap shows the DNA methylation levels of individual CpG sites as measured by bisulfite sequencing. (**C**) Bisulfite sequencing of *cGAS* CpG island in CT1BA5-EC and HSR cells after 10 μM decitabine or DMSO treatment for 48 hrs.

Figure S5. The CT1BA5 tumors are resistant to targeted therapy and immune checkpoint blockade but are sensitive to pharmacological activation of the cytosolic DNA sensing pathway. (**A**) Growth curves of CT1BA5-EC, CT1BA5-HSR and BMFA3 subcutaneous tumors in C57BL/6 (n=7-10) and NSG mice (n=6). (**B**) Growth curves of CT1BA5-EC and CT1BA5-HSR subcutaneous tumors after intraperitoneal treatment with anti-PD-L1 (200 μg) or anti-CTLA4 (100 μg) antibody at indicated times (n=4-6) (**C**) Growth curves of CT1BA5-EC, CT1BA5-HSR and BMFA3 subcutaneous tumors after intratumoral injections of MRTX1133 (30 mg/kg) twice a day. (**D**) Growth curves of CT1BA5-EC and CT1BA5-HSR subcutaneous tumors after intratumoral injections of cGAMP (10 μg) (n=4-6).

Figure S6. Supporting data for the roles of cGAS-STING pathway in restricting ecDNA formation. (**A**) Immunoblotts show protein levels of cGAS and STING in human HAP1 cells. (**B**) HAP1 cell lines were stably transduced with wild-type *STING* (WT) or the cGAMP-binding-deficient R238A/Y240A mutant (mut). Cells were transfected with HT-DNA for 6 hrs and the levels of *IFNβ* transcript were quantified by RT-qPCR. (**C**) Immunoblots show levels of cGAS and IRF3 after CRISPR-mediated knockdown in HAP1 cells.

Figure S1

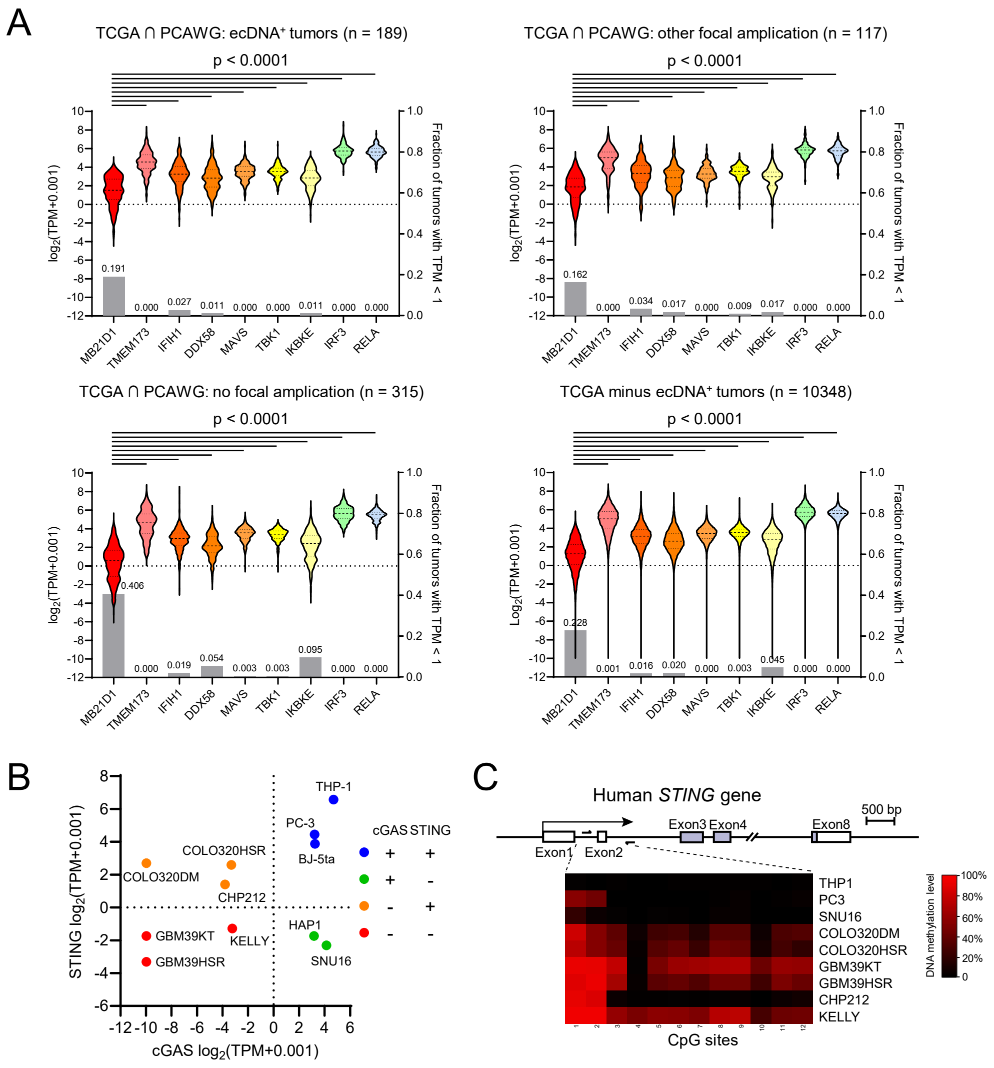
A
TCGA ∩ PCAWG: ecDNA⁺ tumors (n = 189)
TCGA ∩ PCAWG: other focal amplication (n = 117)
TCGA ∩ PCAWG: no focal amplication (n = 315)
TCGA minus ecDNA⁺ tumors (n = 10348)
p < 0.0001
log2(TPM+0.001)
Fraction of tumors with TPM < 1
MB21D1
TMEM173
IFIH1
DDX58
MAVS
TBK1
IKBKE
IRF3
RELA
B
STING log2(TPM+0.001)
cGAS log2(TPM+0.001)
THP-1
PC-3
BJ-5ta
COLO320HSR
COLO320DM
CHP212
GBM39KT
GBM39HSR
KELLY
HAP1
SNU16
cGAS STING
C
Human STING gene
500 bp
Exon1
Exon2
Exon3
Exon4
Exon8
THP1
PC3
SNU16
COLO320DM
COLO320HSR
GBM39KT
GBM39HSR
CHP212
KELLY
CpG sites
DNA methylation level

Figure S2

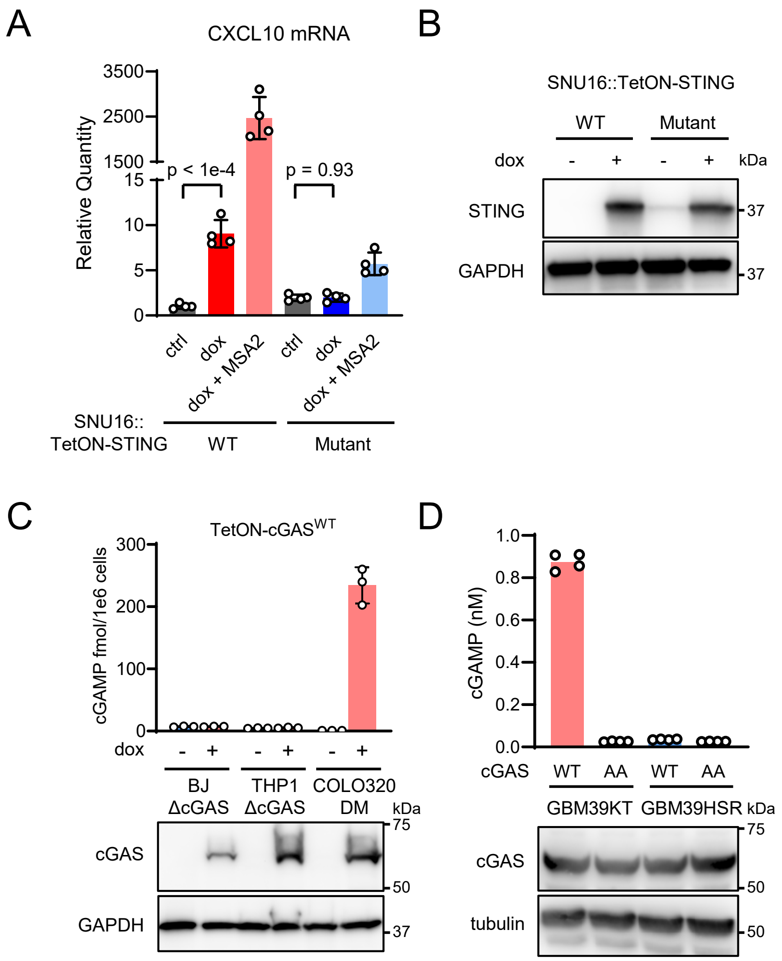

A
CXCL10 mRNA
Relative Quantity
3500
2500
1500
15
10
5
0
p < 1e-4
p = 0.93
ctrl
dox
dox + MSA2
ctrl
dox
dox + MSA2
SNU16::
TetON-STING
WT
Mutant
B
SNU16::TetON-STING
WT
Mutant
dox
-
+
-
+
kDa
STING
GAPDH
37
37
C
TetON-cGAS^WT
cGAMP fmol/1e6 cells
300
200
100
0
dox
-
+
-
+
-
+
BJ
ΔcGAS
THP1
ΔcGAS
COLO320
DM
kDa
cGAS
GAPDH
75
50
37
D
cGAMP (nM)
1.0
0.8
0.6
0.4
0.2
0.0
cGAS
WT
AA
WT
AA
GBM39KT
GBM39HSR
kDa
cGAS
tubulin
75
50
50

Figure S3

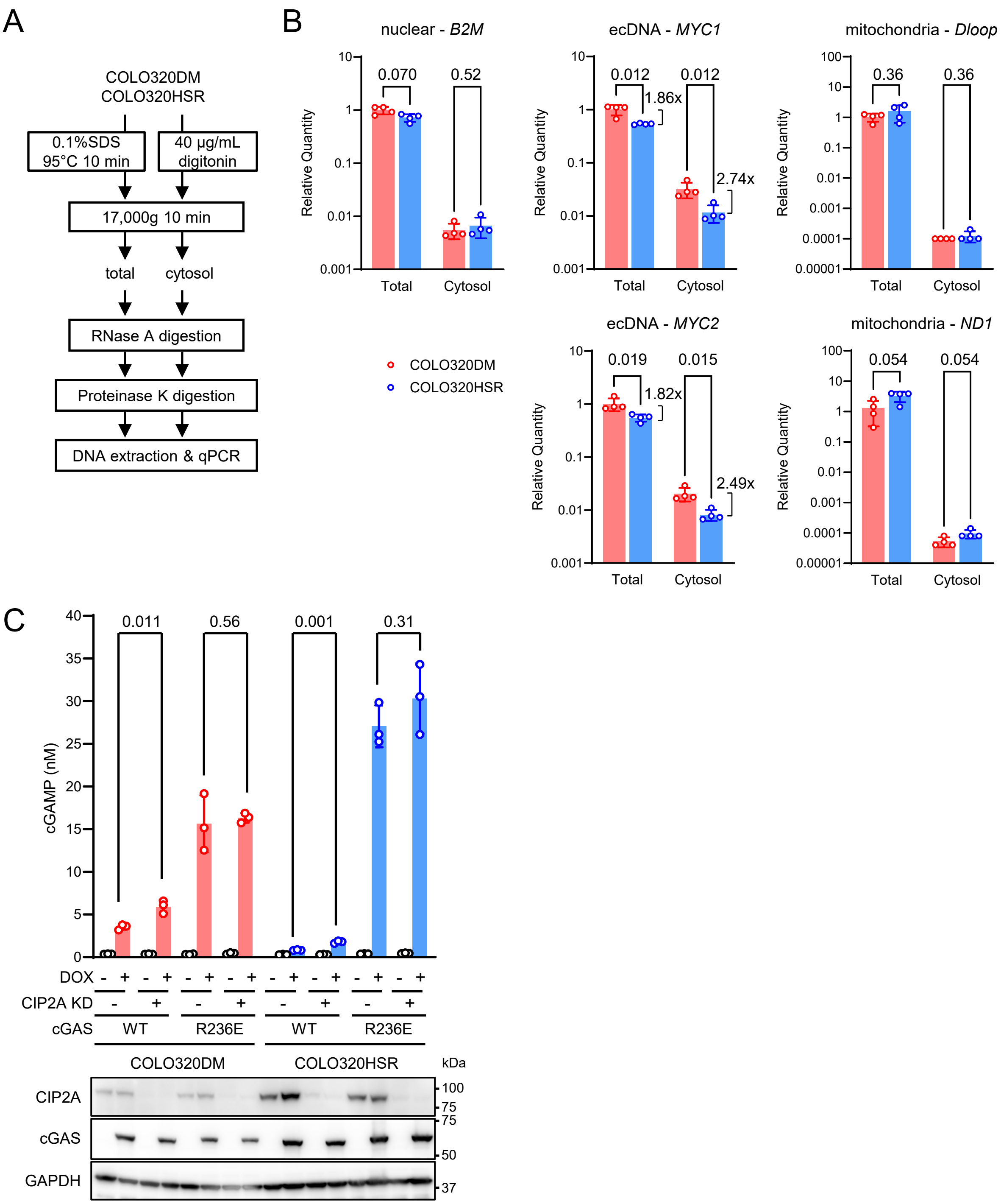
A
COLO320DM
COLO320HSR
0.1%SDS 95°C 10 min
40 µg/mL digitonin
17,000g 10 min
total
cytosol
RNase A digestion
Proteinase K digestion
DNA extraction & qPCR
B
nuclear - B2M
ecDNA - MYC1
mitochondria - Dloop
ecDNA - MYC2
mitochondria - ND1
Relative Quantity
0.070
0.52
0.012
0.012
1.86x
2.74x
0.36
0.36
0.019
0.015
1.82x
2.49x
0.054
0.054
Total
Cytosol
COLO320DM
COLO320HSR
C
cGAMP (nM)
0.011
0.56
0.001
0.31
DOX
CIP2A KD
cGAS
WT
R236E
WT
R236E
COLO320DM
COLO320HSR
kDa
CIP2A
cGAS
GAPDH
100
75
75
50
37

Figure S4

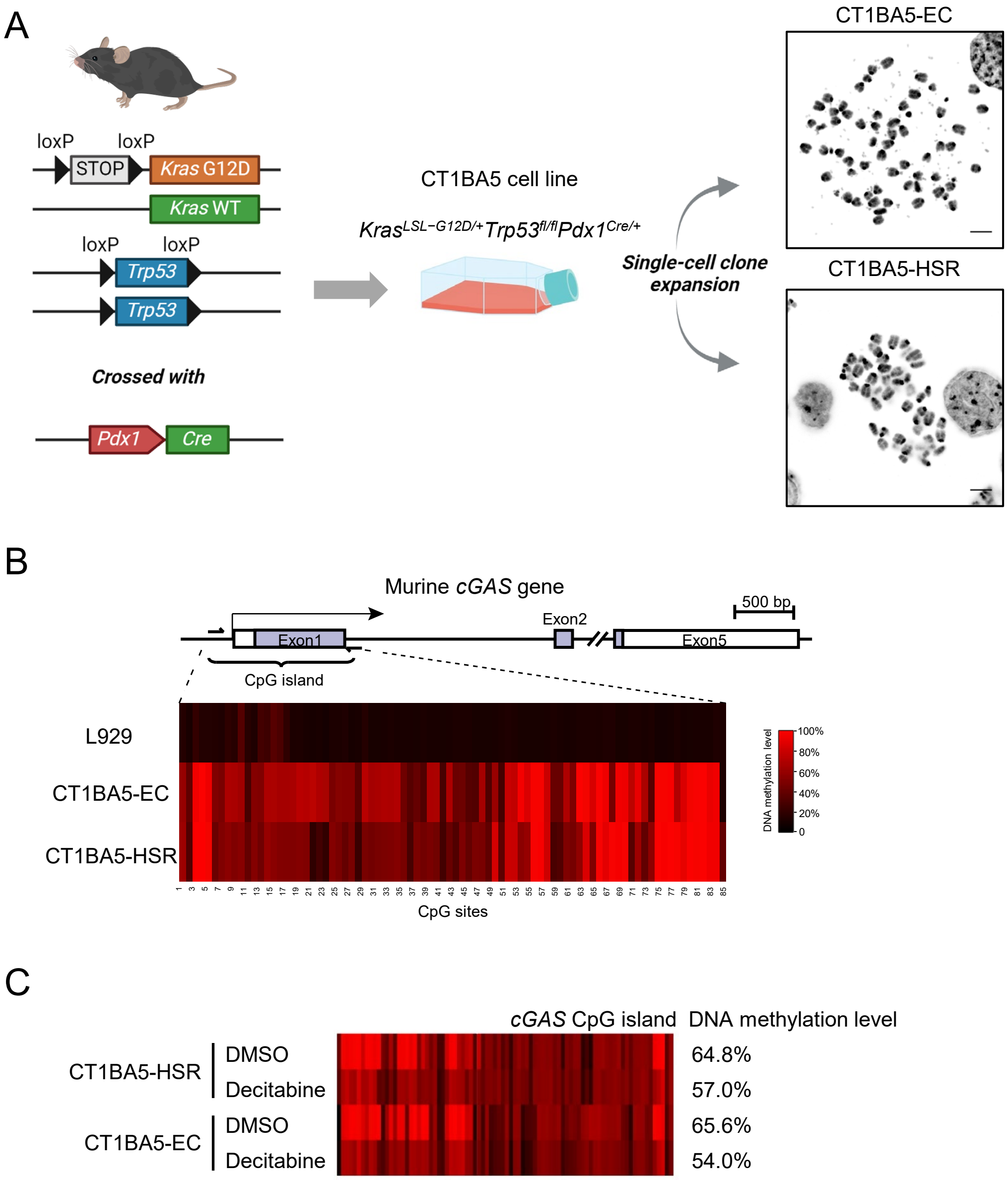
A
loxP
loxP
STOP
Kras G12D
Kras WT
loxP
loxP
Trp53
Trp53
Crossed with
Pdx1
Cre
CT1BA5 cell line
Kras$^{LSL-G12D/+}$Trp53$^{fl/fl}$Pdx1$^{Cre/+}$
Single-cell clone expansion
CT1BA5-EC
CT1BA5-HSR
B
Murine cGAS gene
500 bp
Exon1
Exon2
Exon5
CpG island
L929
CT1BA5-EC
CT1BA5-HSR
CpG sites
DNA methylation level
100%
80%
60%
40%
20%
0
C
cGAS CpG island
DNA methylation level
CT1BA5-HSR
DMSO
Decitabine
CT1BA5-EC
DMSO
Decitabine
64.8%
57.0%
65.6%
54.0%

Figure S5

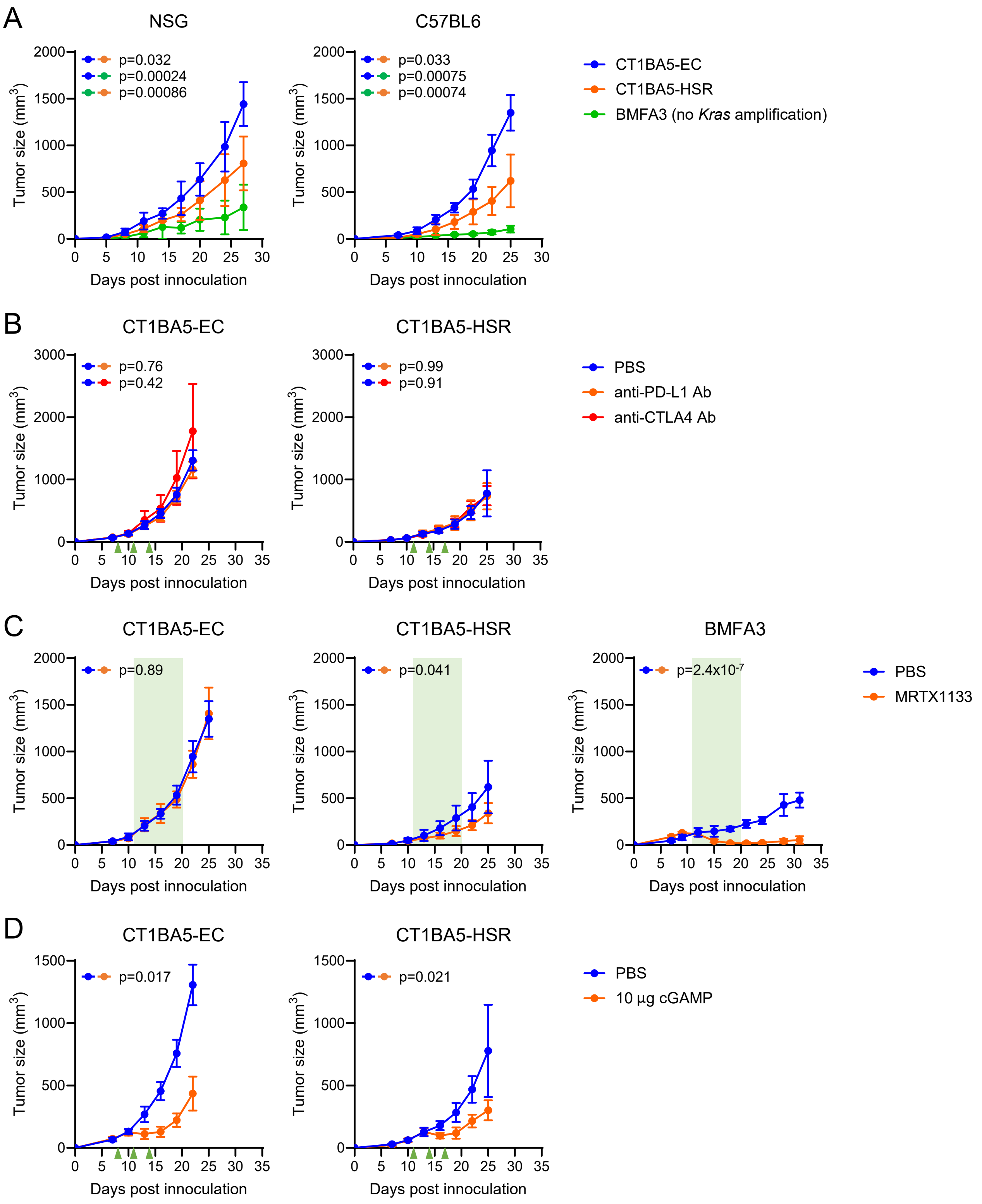

A
NSG
p=0.032
p=0.00024
p=0.00086
C57BL6
p=0.033
p=0.00075
p=0.00074
CT1BA5-EC
CT1BA5-HSR
BMFA3 (no Kras amplification)
B
CT1BA5-EC
p=0.76
p=0.42
CT1BA5-HSR
p=0.99
p=0.91
PBS
anti-PD-L1 Ab
anti-CTLA4 Ab
C
CT1BA5-EC
p=0.89
CT1BA5-HSR
p=0.041
BMFA3
p=2.4x10-7
PBS
MRTX1133
D
CT1BA5-EC
p=0.017
CT1BA5-HSR
p=0.021
PBS
10 µg cGAMP
Tumor size (mm3)
Days post innoculation

Figure S6

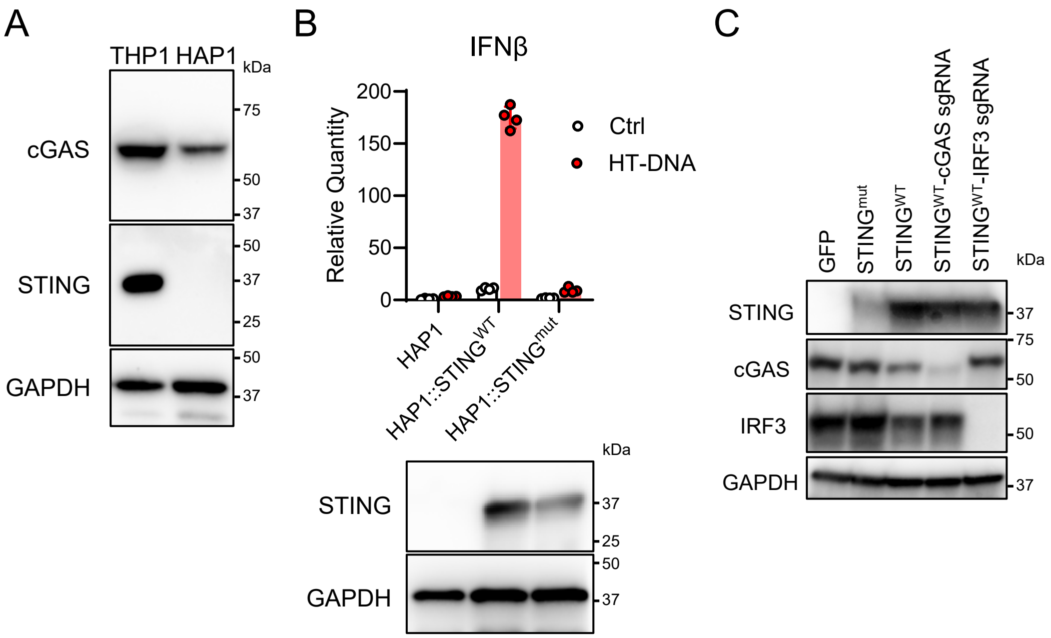
A
THP1
HAP1
kDa
cGAS
75
50
37
STING
50
37
25
GAPDH
50
37
B
IFNβ
Relative Quantity
200
150
100
50
0
Ctrl
HT-DNA
HAP1
HAP1::STING^WT
HAP1::STING^mut
kDa
STING
37
25
GAPDH
50
37
C
GFP
STING^mut
STING^WT
STING^WT-cGAS sgRNA
STING^WT-IRF3 sgRNA
kDa
STING
37
cGAS
75
50
IRF3
50
GAPDH
37
